# Printing High‐Efficiency Perovskite Solar Cells in High‐Humidity Ambient Environment—An In Situ Guided Investigation

**DOI:** 10.1002/advs.202003359

**Published:** 2021-01-25

**Authors:** Patrick Wai‐Keung Fong, Hanlin Hu, Zhiwei Ren, Kuan Liu, Li Cui, Tao Bi, Qiong Liang, Zehan Wu, Jianhua Hao, Gang Li

**Affiliations:** ^1^ Department of Electronic and Information Engineering Research Institute for Smart Energy (RISE) The Hong Kong Polytechnic University Hung Hom Kowloon Hong Kong China; ^2^ The Hong Kong Polytechnic University Shenzhen Research Institute Guangdong Shenzhen 518057 China; ^3^ Hoffmann Institute of Advanced Materials Shenzhen Polytechnic 7098 Liuxian Boulevard Shenzhen 518055 China; ^4^ Department of Applied Physics The Hong Kong Polytechnic University Hong Kong SAR China

**Keywords:** air‐knife assisted drying, blade coating, crystallization, nucleation, perovskite solar cells, scalable ambient fabrication

## Abstract

Extensive studies are conducted on perovskite solar cells (PSCs) with significant performance advances (mainly spin coating techniques), which have encouraged recent efforts on scalable coating techniques for the manufacture of PSCs. However, devices fabricated by blade coating techniques are inferior to state‐of‐the‐art spin‐coated devices because the power conversion efficiency (*PCE*) is highly dependent on the morphology and crystallization kinetics in the controlled environment and the delicate solvent system engineering. In this study, based on the widely studied perovskite solution system dimethylformamide–dimethyl sulfoxide, air‐knife‐assisted ambient fabrication of PSCs at a high relative humidity of 55 ± 5% is reported. In‐depth time‐resolved UV–vis spectrometry is carried out to investigate the impact of solvent removal and crystallization rate, which are critical factors influencing the crystallization kinetics and morphology because of adventitious moisture. UV–vis spectrometry enables accurate determination of the thickness of the wet precursor film. Anti‐solvent‐free, high‐humidity ambient coatings of hysteresis‐free PSCs with *PCE*s of 21.1% and 18.0% are demonstrated for 0.06 and 1 cm^2^ devices, respectively. These PSCs exhibit comparable stability to those fabricated in a glovebox, thus demonstrating their high potential.

## Graphical abstract

In this work, a perovskite solar cell (PSC) is fabricated via a scalable solution process under ambient conditions. A hysteresis‐free, high‐efficiency (21.1%), laminar‐air‐knife‐assisted meniscus coating of PSCs is demonstrated at room temperature and a relative humidity of 55%. An in‐depth in situ investigation by time‐resolved UV–vis spectroscopy is conduced to explore the nucleation dynamics.

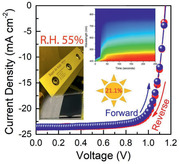

## 1 Introduction

Hybrid organic–inorganic perovskite solar cells (PSCs) have emerged as promising photovoltaic (PV) candidates for commercialization and have attracted significant attention owing to their high light‐to‐electricity power conversion efficiency (*PCE*), low‐cost fabrication, and flexible mechanical properties.^[^ ^1^, ^2^, ^3^, ^4^ ^]^ Perovskites have a general chemical formula of AMX_3_, in which the M metallic cations and X anions form MX_6_^4−^ octahedra with the A cations occupying the 12‐fold coordination holes within the cavity. These materials exhibit desirable optical‐electrical properties for PV devices, such as suitable and tunable bandgap, strong optical absorption, long carrier diffusion length, and high defect tolerance.^[^ ^5^, ^6^ ^]^ A maximum certified *PCE* of 25.2% has been reported for a small‐area PSC (<0.1 cm^2^).^[^ ^7^ ^]^ Despite the remarkable progress in PSCs, several technical and fundamental challenges have to be overcome for their applications, such as the scaling‐up issue,^[^ ^8^ ^]^ long‐term stability under operational conditions,^[^ ^9^ ^]^ and low‐cost mass‐manufacture^[^ ^10^ ^]^ of efficient PSCs. Extensive studies have been conducted on scalable fabrication, including the widely used meniscus coatings^[^ ^11^, ^12^ ^]^ (blade coating, wire‐bar coating, and slot‐die coating) as well as inkjet printing,^[^ ^13^ ^]^ screen printing,^[^ ^14^ ^]^ electrodeposition,^[^ ^10^ ^]^ and vapor‐phase deposition^[^ ^15^ ^]^ to fabricate perovskite light‐harvesting layers. These techniques result in small‐area devices with *PCE*s comparable to those of devices fabricated by spin coating. Recently, a perovskite module with a certified *PCE* of 17.9% and area >19 cm^2^ has been demonstrated via scalable fabrication.^[^ ^16^ ^]^ Significant efforts have been made to address the stability issue, such as perovskite composition engineering,^[^ ^2^, ^17^ ^]^ perovskite light‐harvesting layer passivation,^[^ ^3^, ^18^ ^]^ additive incorporation,^[^ ^19^ ^]^ highly stable charge‐transport material applications,^[^ ^20^ ^]^ interface engineering,^[^ ^21^ ^]^ and encapsulation,^[^ ^22^ ^]^ which have considerably improved the stability of PSCs in both thermal and ambient conditions (anti‐water and anti‐oxygen).

**Figure 1:**
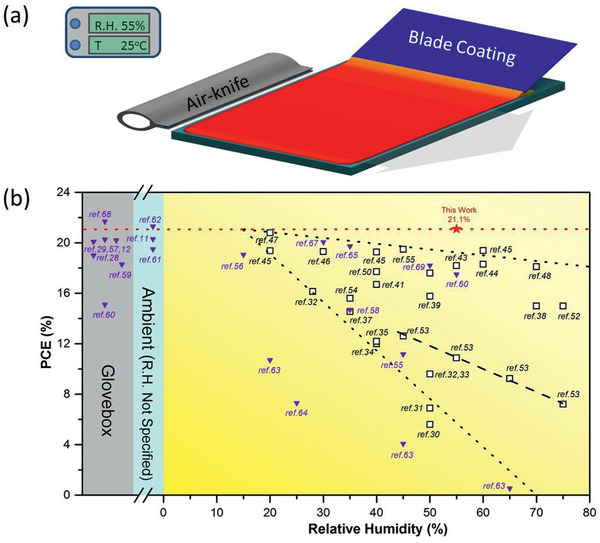
a) Schematic illustration of room‐temperature air‐knife‐assisted blading of a perovskite light‐harvesting layer in air with relative humidity of 55% (this study). b) Representative *PCE* of PSCs fabricated by a spin coating method (open symbol), and blade coating method (solid symbol) under different relative humidities.

Along with the requirement of high PV performance of PSCs, perovskite PV technology demands a low‐cost production approach to achieve sustainability for large‐scale applications in the near future.^[^ ^23^, ^24^ ^]^ However, for highly efficient PSCs fabricated by spin coating^[^ ^25^, ^26^, ^27^ ^]^ or blade coating,^[^ ^12^, ^28^, ^29^ ^]^ an inert gas‐filled glovebox is typically employed during fabrication. This may lead to an external cost barrier,^[^ ^10^ ^]^ which can significantly impede large‐scale industrial fabrication and hamper the application of perovskite PV technology. Therefore, a simple and ambient air‐processed fabrication approach is required for industrial manufacturing. Studies have been conducted on the ambient air‐processed perovskite layer using the spin coating method^[^ ^30^, ^31^, ^32^, ^33^, ^34^, ^35^, ^36^, ^37^, ^38^, ^39^, ^40^, ^41^, ^42^, ^43^, ^44^, ^45^, ^46^, ^47^, ^48^, ^49^, ^50^, ^51^, ^52^, ^53^, ^54^, ^55^ ^]^ and the blade coating method,^[^ ^11^, ^12^, ^28^, ^29^, ^55^, ^56^, ^57^, ^58^, ^59^, ^60^, ^61^, ^62^, ^63^, ^64^, ^65^, ^66^, ^67^, ^68^, ^69^ ^]^ as shown in **Figure** **1**a. For example, to fabricate PSCs possessing high *PCE*s and long‐term stabilities under ambient conditions, the incorporation of Cs was found to significantly affect the grain size and morphology of the Cs/MA/FA perovskite layer processed in ambient air with a relative humidity of 20%.^[^ ^47^ ^]^ However, as the relative humidity of the fabrication process increases, the *PCE* of the PSC decreases, as indicated by the two black dotted lines in Figure 1b. The additive 4‐tert‐butylpyridine (tBP) has been used as a morphology‐modifying agent to improve the performance of PSCs.^[^ ^70^, ^71^ ^]^ The hydrophobic end of tBP is believed to significantly improve the moisture resistance of the perovskite layer.^[^ ^53^ ^]^ Besides additive‐assisted fabrication, high‐temperature and hot‐solution blading in air has also been explored.^[^ ^11^, ^60^, ^61^, ^62^, ^72^ ^]^ Room‐temperature fabrication in ambient air conditions is highly desired for the industrial application of perovskite PV technology. To obtain a high‐quality, pinhole‐free perovskite light‐harvesting layer, gas‐blow‐assisted drying has been adopted to control the nucleation during the perovskite thin‐film formation process, as shown in Figure 1a.

MAPbI_3_ is widely used as a light‐harvesting layer in PSCs. Pioneering work based on blade‐coated MAPbI_3_ was conducted by Huang et al.^[^ ^65^ ^]^ They used ammonium chloride as an additive in the MAPbI_3_ precursor solution to suppress the PbI_2_ phase and to reduce the trap density to fabricate MAPbI_3_‐based perovskite solar modules on flexible glass substrates, and reported a record efficiency of 15.86% on modules with an area of 42.9 cm^2^.^[^ ^65^ ^]^ De‐wetting of the perovskite precursor solution is an obstacle in the blade coating method, particularly for hydrophobic poly(triaryl amine)‐coated indium tin oxide substrates. A surfactant was used to improve the wetting of the precursor solution to achieve a high blading speed of 50 mm s^−1^ at a substrate temperature of 145 °C.^[^ ^11^ ^]^ Room‐temperature, high‐speed (99 mm s^−1^) blade coating of MAPbI_3_ was realized by tailoring the solvent, resulting in a certified stabilized *PCE* of 16.4% for a perovskite module with an area of 63.7 cm^2^.^[^ ^62^ ^]^ Liu et al. demonstrated a significant improvement in the stability of perovskite films and devices under moisture, thermal, and light‐soaking conditions by incorporating a multifunctional sulfobetaine‐based zwitterionic surfactant into the ink for room‐temperature blade coating of PSCs.^[^ ^73^ ^]^ Recent studies have also described meniscus blade coating of perovskite films by incorporating thiourea into precursor solution to facilitate the coating of a compact perovskite layer on a rough surface in a glovebox,^[^ ^74^ ^]^ incorporating organic halide molecules in FA‐alloyed PSCs to enhance phase purity and stability,^[^ ^75^ ^]^ and two‐step sequential blade coating of flexible PSCs with a record fill factor (*FF*) of 81% under ambient conditions with 20% relative humidity.^[^ ^76^ ^]^ Air‐knife‐assisted, room‐temperature blade‐coated PSCs have been reported by our group (*PCE* of 20.26%, using an inert gas glovebox),^[^ ^12^ ^]^ and Huang et al. (*PCE* up to 21.3%, using delicate solvent engineering in ambient air with an unspecified relative humidity),^[^ ^62^ ^]^ indicating high potential for large‐scale applications. A majority of efficient PSCs have been developed in dimethylformamide–dimethyl sulfoxide (DMF–DMSO)‐based solvent systems; therefore, using the air‐knife‐assisted perovskite film coating technique on these inks to achieve high PSC performance in ambient air conditions, particularly in a relatively high‐humidity environment, is highly desired.

In this study, we performed systematic investigations on the fabrication of PSCs at room temperature in ambient air conditions with relatively high humidity (*RH* = 55 ± 5%) using a DMF–DMSO‐based solvent system. Real‐time UV–vis spectrometry measurements were conducted to elucidate the impact of the air‐knife (N_2_)‐assisted blading process on the drying kinetics and crystallization of perovskite thin films. The UV–vis spectrometry data were analyzed for the determination of wet precursor film thickness, which is important for coating quality control; the obtained information can be applied in the manufacturing process to enable reliable thickness control under different environmental conditions. Combined with morphological and photoluminescence (PL) characterization, our results indicate that air‐knife‐assisted drying significantly contributed to nucleation of the perovskite light‐harvesting layer, determination of the thin film morphology and PV performance, and protection of the perovskite and its intermediate phase against moisture during solidification. To elucidate the nucleation and crystal growth mechanism of perovskite thin films in ambient air conditions, the air‐knife blowing velocity was varied systematically to examine the perovskite thin‐film nucleation mechanism using the LaMer model. With this robust ambient coating method, we fabricated hysteresis‐free PSCs with *PCE*s of 21.1% and 18.0% for small‐area (0.06 cm^2^) and large‐area (1 cm^2^) devices, respectively. Notably, the *PCE* of the ambient‐air‐processed blade‐coated device is comparable to that of the glovebox‐processed device, which is supported by PL characterization. Our results demonstrate a promising laboratory‐scale glovebox process that can be efficiently applied in ambient‐air manufacturing.

## 2 Results and Discussion

Meniscus coating is a facile, simple coating method that is compatible with industrial‐scale manufacturing process. This coating technique includes dip coating, blade coating, and slot‐die coating. Recent studies have demonstrated the use of meniscus‐blade coating technique followed by air‐knife‐assisted drying to achieve highly efficient PSCs prepared in an inert‐gas‐filled glovebox,^[^ ^11^, ^12^, ^62^, ^68^ ^]^ indicating a laboratory‐based fabrication technology (spin coating) that can be scaled‐up to form an industrial‐scale manufacturing process. In this study, the meniscus‐blade coating technique was used to prepare wet perovskite films on a 30 nm SnO_2_‐coated fluorine‐doped tin oxide (FTO) substrate in ambient air with a relative humidity of 55 ± 5%, as shown in Figure 1a. We adopted a mixed‐cation and mixed‐anion perovskite precursor (MAPbBr_3_)_0.15_ (FAPbI_3_)_0.85_ containing 5% Cs for one‐step blade coating at room temperature of 25 °C. A small amount of precursor solution was injected into the gap between the blade and substrate to initiate the static wetting process. The precursor solution rapidly spread along the edge of the blade in a uniform manner, which was attributed to the capillary effect in the small gap between the blade and the substrate to form a wetting line. The gap spacing between the blade and the substrate was fixed at 90 µm throughout this study. The wetting line was moved laterally with respect to the substrate surface at a velocity of 10 mm·s^−1^, resulting in a uniform perovskite precursor wet film.

**Figure 2:**
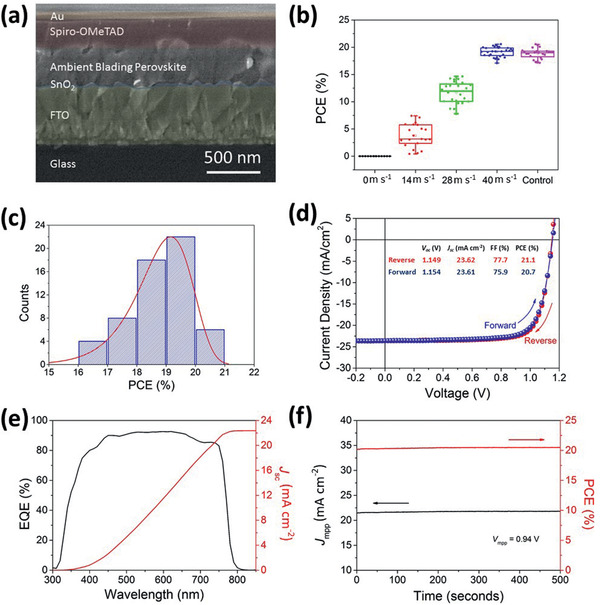
a) Cross‐sectional SEM image of ambient‐air‐blade‐coated PSC with air‐knife blowing at 40 m s^−1^, and b) *PCE* distribution as a function of air‐knife blowing velocity of the film prepared in ambient air and the control prepared in glovebox. c) *PCE* distribution histogram of 59 ambient‐air‐processed devices. d) *J–V* characteristics, e) *EQE* spectrum and the corresponding integrated *J*_SC_, and f) stability test at the maximum power point of the champion device with an area of 0.06 cm^2^.

To realize successful manufacturing, along with the development of scalable techniques, a better understanding of the environmental challenges that accompany fabrication, particularly the moisture attack during scalable deposition in ambient air conditions, is necessary. The most challenging problem of solution‐processed organic–inorganic perovskite thin films in ambient air is the regulation of the density of nucleation sites and the crystallization process, which was controlled by the air‐knife gas flow rate/solvent removal rate in this study. Herein, we elucidate the perovskite solution‐to‐solid crystallization mechanism in ambient air with 55% relative humidity through extensive in situ thin‐film examination, building connections with crystal growth models, thin films, and device characterizations. We begin by evaluating the PV performance of the air‐processed meniscus‐blade‐coated perovskite photo‐harvesting layer with different air‐knife‐assisted drying gas velocities (0, 14, 28, and 40 m s^−1^). The fabrication of planar n‐i‐p PSCs was based on the glass/FTO/SnO_2_/ambient‐air‐coated perovskite/spiro‐OMeTAD/Au. The corresponding cross‐sectional scanning electron microscopy (SEM) image is shown in **Figure** **2**a. Figure 2b shows the statistical PV performance of devices fabricated with the ambient air‐processed perovskite photo‐harvesting layer dried at different air‐knife gas velocities. In case of the perovskite thin‐film dried in ambient air without the use of an air‐knife, that is being dried naturally, no PV effect occurs, which is attributed to the extremely poor film coverage and crystallinity. It is well‐known that controlling perovskite nucleation and crystallization is the key to achieving high‐quality perovskite thin‐films by modulating the film morphology and elongating carrier lifetime.^[^ ^1^, ^12^, ^36^, ^77^ ^]^ We systematically investigated the influence of different solvent drying rates on the optoelectronic quality of perovskite thin‐films using air‐knife‐assisted drying on an ambient‐air‐blade‐coated wet film. It was observed that the average *PCE* increases and scattering in *PCE* decreases as the air‐knife‐assisted blowing velocity increases, as shown in Figure 2b. At low air‐knife blowing velocity (14 and 28 m s^−1^), the poor PV parameters (summarized in Table S1, Supporting Information)—open‐circuit voltage (*V*_OC_), short‐circuit current density (*J*_SC_), and *FF*—are attributed to the uncovered regime and pinholes in the perovskite films,^[^ ^12^ ^]^ which result in pathways between the electron‐ and hole‐transport layers, thus enhancing surface charge recombination and deteriorating device performance.^[^ ^78^ ^]^ Notably, the *PCE* distribution of the ambient air‐knife‐processed perovskite thin‐film dried at a gas velocity of 40 m s^−1^ was comparable to that of the glovebox air‐knife‐processed (control) thin film, as shown in Figure 2b. Figure 2c shows the statistical *PCE* distribution of a batch of 59 ambient‐air‐processed (blade‐coated and air‐knife‐assisted drying at 40 m s^−1^) perovskite devices with an average *PCE* of over 19%. Figure 2d shows the current–voltage (*J–V*) characteristics of the champion device fabricated in ambient air with a relative humidity of 55 ± 5%. The highest *PCE* achieved from the reverse scan was 21.1% with a *V*_OC_ of 1.15 V, *J*_SC_ of 23.62 mA cm^−2^, and *FF* of 0.78. Negligible hysteresis was observed by comparing the *J–V* curves obtained from the reverse and forward scans. The detailed PV parameters are shown in the inset of Figure 2d. To the best of our knowledge, the *PCE* achieved in this work is one of the best values achieved by air‐processed, blade‐coated PSCs,^[^ ^62^ ^]^ as shown in Figure 1b. External quantum efficiency (*EQE*) measurements were also performed on the champion device to further confirm the *J*_SC_, as shown in Figure 2e. The integrated *J*_SC_ (22.34 mA cm^−2^) obtained from the *EQE* curve agrees well with the *J*_SC_ estimated from the *J–V* measurement (23.62 mA cm^−2^), showing a 5% deviation. Furthermore, the *EQE* spectrum shows a strong photoresponse (the highest value from the *EQE* profile is over 90%), indicating a high‐quality perovskite photo‐harvesting layer and a good connection of the stacked device.^[^ ^79^ ^]^ Figure 2f shows the stabilized photocurrent of the champion device at the maximum power output bias (0.94 V) and the calculated *PCE* under one sun illumination. The photocurrent was stabilized at 21.85 mA cm^−2^, yielding a decently stabilized *PCE* of 20.6%. The shelf‐life stability of unsealed 0.06 cm^2^ devices was measured over 80 days, as shown in Figure S1, Supporting Information. Our results indicate ≈5% reduction in *PCE* for the ambient‐air‐processed and glovebox‐processed devices after 80 days. Most importantly, the ambient‐air‐processed devices exhibit device performance and stability comparable to that of the glovebox‐processed devices.

We fabricated highly efficient PSCs with *PCE* up to 21.1% with the perovskite photo‐harvesting layer prepared in ambient air using a meniscus‐blade‐coated technique with air‐knife‐assisted drying at an optimal drying gas velocity of 40 m s^−1^. The bladed‐coated precursor wet film was subjected to solvent evaporation, intermediate (solvate) solid‐state film, and solidification processes to form a high‐quality perovskite thin film. Each of these steps plays an important role in determining the morphology, electrical, and optical quality of the dried crystalline perovskite thin film. Several approaches have been considered to control the solvent removal process, such as anti‐solvent dripping, gas quenching, and hot‐cast coating. However, solvent dripping is not applicable in either ambient spin coating or blade coating of the perovskite precursor solution. The hot‐solution‐casted coating process is highly dynamic, and it is difficult to control and conduct detailed studies on the same. Air‐knife gas quenching (using an inert gas such as nitrogen)^[^ ^12^, ^62^, ^80^ ^]^ was adopted in our study.

**Figure 3:**
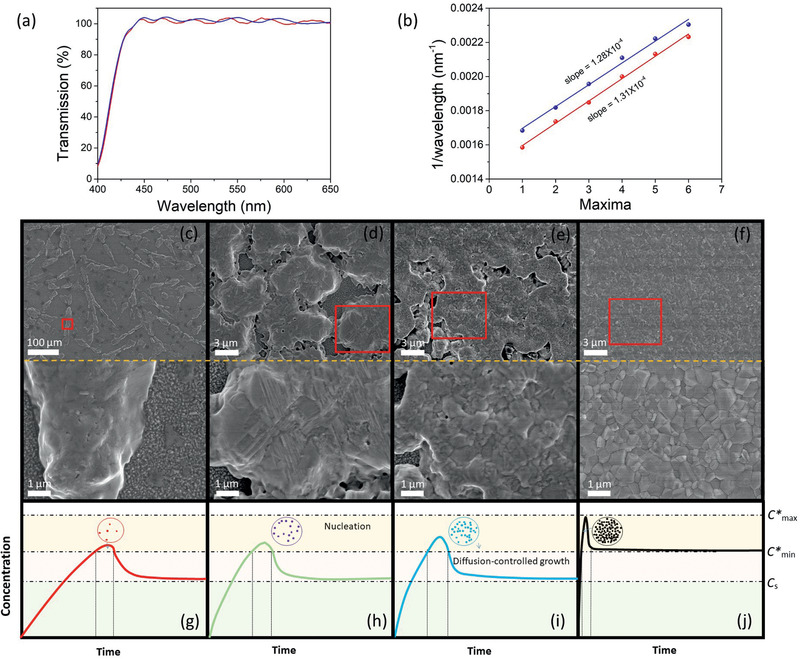
a) In situ UV–vis transmission spectra of the as‐bladed precursor wet film in ambient air from two different samples. b) Plot of 1/*λ* versus maxima order extracted from the transmission spectrum exhibiting interference oscillations. c–f) Top‐view SEM images of perovskite thin‐films fabricated upon different blowing rates (0, 14, 28, and 40 m s^−1^) and the associated images of higher magnification in the area marked by the red rectangle. g–j) Illustration of different nucleation and perovskite crystal growth behavior under varied blowing rates based on the classical LaMer model.

In situ UV–vis spectrometry is a powerful tool to monitor the coating thickness of the wet film. It was observed that the as‐bladed wet film exhibited a sinusoidal interference pattern superimposed on the transmission spectrum, indicating a uniform and smooth wet film surface, as shown in **Figure** **3**a. The thickness of the wet perovskite film can be compared by a simple slope fitting or evaluated from a detailed calculation. Constructive interference occurs at the condition 2*n*_f_ *d*_f_ = (*m* + 1/2) *λ*, where *n*_f_, *d*_f_, and *m* are the refractive index of the wet film, thickness of the wet film, and maxima order, respectively. Thus, the slope of a plot of 1/*λ* versus the maxima order is equal to 1/(2*n*_f_ *d*_f_). Although *n*_f_ and *d*_f_ are both unknown, nearly the same slope is obtained from two different wet films, as shown in Figure 3b, indicating equal thickness of the two independent wet films bladed in an ambient air environment. Thus, the ambient air‐blade‐coated wet films are flat and highly reproducible based on our in situ UV–vis spectrometry analysis. This is an efficient and effective method for monitoring the first parameter of the blade coating process: the thickness of the as‐bladed wet film. We believe that this is beneficial to the manufacturing process because the thickness of the perovskite precursor wet film can be precisely monitored. Apart from a simple slope comparison, the wet film thickness can be equivalently evaluated from a detailed envelope modeling of the sinusoidal interference pattern.^[^ ^81^ ^]^ Details of the calculation of the thickness of the wet film are given in Note 2, Supporting Information.

We then examined the natural drying process of the meniscus‐blade‐coated perovskite precursor wet film in ambient air conditions with a relative humidity of 55 ± 5% at room temperature (25 °C). Drying of the wet film naturally (without the application of air‐knife‐assisted drying) occurred in 20 min, and small crystallites were formed, resulting in a highly rough surface. Figure S2a, Supporting Information shows the wet film dried naturally under ambient air conditions. The naturally dried wet film exhibits extremely poor film coverage, as shown in Figure S2a, Supporting Information. To further investigate the morphology of the naturally dried sample, SEM analysis was performed. Figure 3c shows the top‐view SEM image of the naturally dried sample. Disordered needle‐like crystallites are formed, and poor film coverage significantly affects the fabrication of solar cells.^[^ ^49^, ^50^, ^82^ ^]^ The poor film coverage results in zero photoresponse, as shown in Figure 2b. Notably, needle‐like crystallites were not observed in perovskite films naturally dried in an N_2_‐filled glovebox in our previous study;^[^ ^12^ ^]^ thus, the formation of these crystal structures is expected to be an ambient‐air‐related phenomenon, most likely due to humidity.

The intrinsic low rate of nucleation in solution‐processed perovskites is due to the slow solvent evaporation rate. The drying kinetics is critical in determining the final morphology of the perovskite active layer as well as the device performance. To form a compact and pinhole‐free active layer, we examined the influence of different gas flow rates on the drying kinetics, which is a direct measure of the rate of nucleation. We used three different N_2_ gas blowing velocities: 14, 28, and 42 m s^−1^ for air‐knife‐assisted solidification. It was found that for the as‐bladed wet film subjected to a low blowing velocity of 14 m s^−1^, the blow accelerated the solvent evaporation rate, which in turn increased the nucleation rate. As shown in Figure 3d, a crystal domain size of ≈10 µm is observed, and the film coverage significantly increases compared with that of the sample without air‐knife‐assisted drying. Thus, the abrupt change in film morphology reveals that air‐knife‐assisted drying plays an important role in the nucleation rate. However, the density of nucleation centers was relatively low for achieving a compact and pinhole‐free perovskite layer. The co‐existence of poor film coverage and pinholes in the perovskite thin‐film after air‐knife‐assisted solvent removal at low blowing velocity is detrimental to device performance. Next, the N_2_ gas blowing velocity was increased to 28 m s^−1^. The film coverage improved, and compact crystallites with sizes of several hundred nanometers are observed, as shown in Figure 3e; the corresponding surface was not sufficiently smooth to provide a good platform for the fabrication of high‐quality PSCs. Rapid solvent removal using a high gas‐blowing speed was found to be critical for fabricating a compact, uniform, and pinhole‐free perovskite thin‐film in an ambient environment, as shown in Figure 3f. At high gas‐blowing speeds, the solvent evaporates quickly, and a high density of nuclei is formed. The nuclei subsequently grow into grains hundreds of nanometers in size. Thus, a systematic trend for improving film morphology was observed: the increase in air‐knife blowing velocity results in an increased PSC performance, as shown in Figure 2b. However, the air‐knife blow speed is not the faster the better, as too large blow force will negatively affect the liquid film to be dried.^[^ ^12^ ^]^ A blow velocity of ≈50 m s^−1^ ensures a sufficiently large blow force, which spreads the wet precursor film across the substrate, resulting in a heterogeneous precursor solid film, as shown in Figure S3, Supporting Information. Scale‐like features are observed, indicative of the solution flow at large blow forces. A similar phenomenon was reported in an air‐bladed perovskite layer, in which a large gas blow force was adopted to spread the perovskite precursor solution on the substrate.^[^ ^57^ ^]^ A lower concentration (36 wt% solute:solution) of the precursor solution was also examined, and a similar trend was observed (Figure S3, Supporting Information).

The classical theories for nanocrystal nucleation and growth have been reviewed elsewhere.^[^ ^83^ ^]^ The growth process can be categorized into two: diffusion‐controlled growth and surface‐reaction‐controlled growth. The growth of perovskite and its intermediate belongs to diffusion‐controlled growth.^[^ ^24^ ^]^ This implies that when the perovskite precursor concentration is below the minimum concentration for nucleation (C*_min_), the perovskite or its intermediate crystal growth continues while the nucleation process stops. To better explain the nanocrystal nucleation and growth from the solution state, a classic LaMer model was considered,^[^ ^84^ ^]^ which typically comprises three parts: i) increase of the growth species in the solution, ii) nucleation in the solution when the growth species concentration exceeds the minimum super‐saturation limit, and iii) crystal growth by the control of growth species diffusion. Each part is indicated with the help of two black dashed lines in Figure 3g–j. The LaMer model separates the nucleation and growth into two steps, with crystal growth controlled by the growth species diffusion. The model links the degree of supersaturation with nucleation and crystal growth, which directly determines the perovskite thin‐film morphology and consequently, the PV performance. Nitrogen blowing can not only control the drying rate of solvent molecules but also protect the perovskite precursor solution against oxygen and moisture in the air. In contrast to the natural drying process resulting in extremely poor film coverage (Figure 3c), high‐speed nitrogen blowing results in a faster solvent drying rate with a higher degree of super‐saturation, yielding burst nucleation (Figure 3j). This eventually leads to a fully covered perovskite thin‐film with compact perovskite domains. According to the Weimarn theory,^[^ ^85^ ^]^ both nucleation and crystal growth are dependent on the degree of super‐saturation in the solution. The average size of crystal clusters is determined by the trade‐off between the nucleation and crystal growth processes. The nucleation rate is defined as follows:

(1) $$V_{1}=cA\exp\left(\frac{-\Delta G^{*}}{k_{B}T}\right)$$

where *k*_B_ is the Boltzmann constant, *c* is a constant, *A* is a complicated function of the molecular‐level diffusion‐kinetics parameters, *T* is the absolute temperature, and ΔG* is the critical free‐energy of nucleation. For a cluster with spherical shape, ΔG* can be expressed as follows:

(2) $$\Delta G^{*}\;=\frac{16\pi \gamma^{2}\Omega^{3}}{3k_{B}^{\;2}T^{2}\sigma^{2}}$$

where *γ* is the surface free‐energy of the critical cluster, *σ* is the degree of super‐saturation of the solution, and *Ω* is the molecular volume of the crystal. According to Equation (1) and (2), *V*_1_ (nucleation rate) exhibits exponential growth as the degree of super‐saturation increases, that is, *V*_1_ ∝ exp(−1/*σ* ^2^). According to the Burton–Cabrera–Frank theory,^[^ ^83^ ^]^ *V*_2_ (crystal growth rate) is also dependent on *σ*. In comparison with *V*_2_, *V*_1_ is relatively more sensitive to *σ* owing to the exponential relationship. The number of clusters per unit area (*N*) can be described as

(3) $$N=1.1{\left(\frac{V_{1}}{V_{2}}\right)}^{1/2}$$

which is inversely proportional to the cluster size. The nitrogen blowing enables a significantly higher nucleation rate based on Equation (1) and (2). Eventually, it leads to a boosted nucleation density with compact perovskite domains, as shown in Figure 3d.

**Figure 4:**
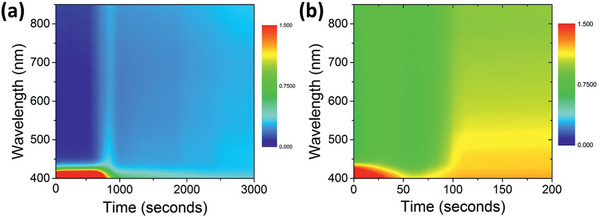
Time‐resolved absorption spectra measured by in situ UV–vis spectrometry for the blade‐coated wet perovskite film sample a) dried naturally and b) thermally dried at 105 °C without gas blowing in ambient conditions. Photographs of the samples are shown in Figure S2a,e, Supporting Information.

A detailed understanding and appropriate control of solidification are critical to successfully fabricate a high‐quality perovskite absorbing layer for PV applications. In this study, in situ time‐resolved (TR) UV–vis spectrometry was used to investigate the drying kinetics of the perovskite precursor solution in detail to understand the drying kinetics of the air‐processed perovskite thin films and identify the bottlenecks that limit the efficiency of the fabricated PSCs. For the drying film‐formation process, we first examined the drying kinetics of the ambient blade‐coated wet film dried naturally. **Figure** **4**a shows TR absorption spectra measured by in situ UV–vis spectrometry for the sample dried naturally at 25 °C in a relative humidity of 55%. The as‐bladed wet film exhibits a strong UV absorption at wavelengths shorter than 450 nm, which is attributed to the solvent complex. The static absorption spectrum of the perovskite precursor solution measured using the same in situ UV–vis spectrometry technique is shown in Figure S4, Supporting Information; it demonstrates absorption similar to that of the as‐bladed wet film. The wet film was subsequently dried naturally in ambient air, and in situ TR UV–vis spectrometry was performed to study the drying kinetics. The solvent complex evaporated almost completely at *t* ≈ 800 s. As shown in Figure 4b, when the as‐bladed wet film was baked immediately at 105 °C, the time required for drying the solvent complex reduced to ≈50 s. Although the solvent complex was dried naturally, it did not provide sufficient nucleation sites for the formation of perovskite solid film, as confirmed by the low absorbance of around 0.3 for *t* > 2000 s. This was in good agreement with the SEM results, as shown in Figure 3c.

**Figure 5:**
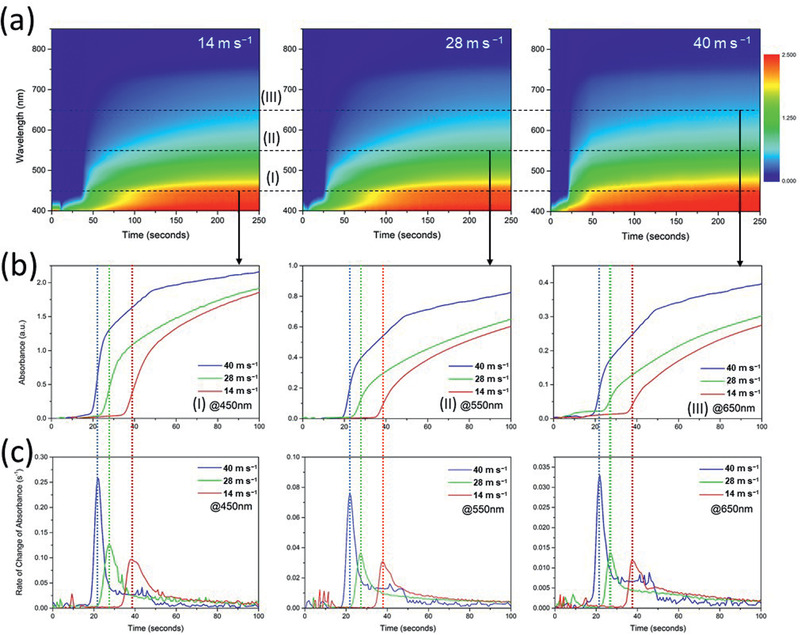
Time‐resolved absorbance spectra measured by in situ UV–vis spectrometry of air‐knife‐assisted drying at a) different gas‐blowing velocities, b) different extracted wavelengths, and c) the corresponding rate of change of absorbance.

We conducted systematic in situ studies on the impact of air‐knife‐assisted drying mechanism by varying the N_2_ gas blowing velocity from 14 to 40 m s^−1^. Three different stages (solution, intermediate, and solid) can be identified from the absorbance spectrum of the in situ TR UV–vis spectrometry, as shown in **Figure** **5**a. The spectra show a similar trend: the solution stage (wet film) exhibits strong UV absorption at wavelengths shorter than 450 nm, and there is zero absorbance at longer wavelengths. Air‐knife‐assisted blowing was applied at *t* = 2 s for each of the samples, which eliminated the solvent complex, and nucleation sites began to form because of the increase in concentration above saturation; this has been discussed using LaMer model, as shown in Figure 3h–j. Thus, the gas blowing assists in removing the solvent, and the intermediate stage starts to appear. A rapid change in absorbance was observed, which is attributed to the formation of perovskite crystallites or the intermediate stage (Lewis acid–base‐type adduct) in the solid precursor film. The absorbance gradually intensified until it reached a stable level, indicating a completely formed solid precursor film. The blowing gas velocity plays an important role not only in avoiding moisture attack but also in the rapid solidification process. As shown in Figure 5a, the time required to remove the solvent complex (accompanied by strong UV absorption below 450 nm) is estimated to be 11, 5, and ≈0 s for blowing velocities of 14, 28, and 40 m s^−1^, respectively, as expected.

To understand the transition mechanism in the intermediate state, in situ TR absorbance data were analyzed at the selected wavelengths 450, 550, and 650 nm, which are marked by dashed lines (I), (II), and (III), respectively, in Figure 5a. Quantitative analysis of the drying kinetics was first conducted by extracting the absorbance at 450, 550, and 650 nm versus time, as shown in Figure 5b. The time required for the rapid increase in absorbance at different wavelengths reduces with higher blowing velocity. The peak absorption change rate is obtained after 38, 27, and 22 s at blowing velocities of 14, 28, and 40 m s^−1^, respectively. The corresponding evolution of the absorbance spectra is shown in Figure S5, Supporting Information. Because the absorbance at the wavelengths 450, 550, and 650 nm represent the absorbances of the solid perovskite film, the first derivative of the absorbance with respect to time was used to quantify the solvent drying rate or crystallization rate. The plots of the first derivatives versus time at different wavelengths are shown in Figure 5c. The highest absorbance change rate was obtained for the sample with N_2_ blown at 40 m·s^−1^. Our calculation shows that the peak absorbance change rate at *λ* = 450 nm, which is a direct parameter quantifying the crystallization rate, is 0.097, 0.127, and 0.259 s^−1^ for gas blowing velocities of 14, 28, and 40 m·s^−1^, respectively. Hence, the crystallization rate for the sample with air‐knife‐assisted drying at a gas‐blowing speed of 40 m s^−1^ is ≈2.7 times higher than that of the sample with a relatively slow blowing speed of 14 m s^−1^. Detailed calculations of the peak absorbance change rate for wavelengths of 550 and 650 nm are summarized in Table S2, Supporting Information. This result is in agreement with the SEM results, which show that slow blowing speed results in a low crystallization rate, resulting in poor film coverage. Thus, in situ TR UV–vis spectrometry is a powerful characterization tool to identify rapid crystallization and quantify the rate of crystallization. This is beneficial for precisely controlling and monitoring thin‐film preparation under ambient conditions.

**Figure 6:**
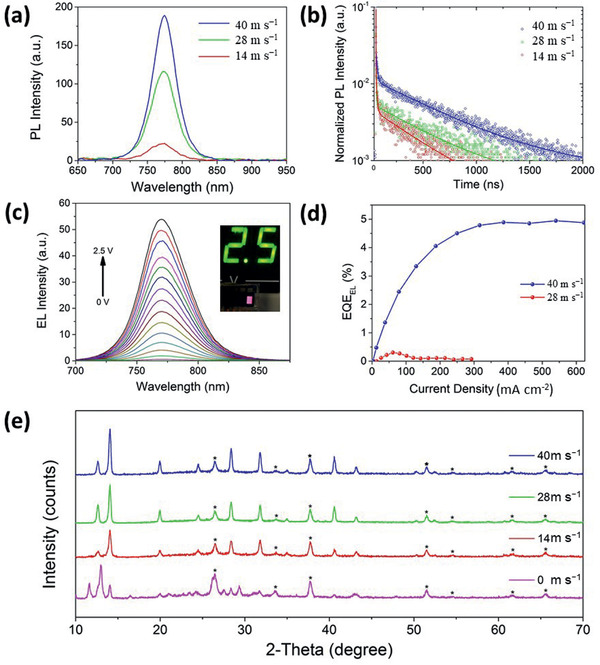
a) Steady‐state and b) TRPL spectra for the samples prepared by air‐knife‐assisted drying at different gas‐blowing velocities. c) EL spectra of the device prepared by air‐knife‐assisted drying at 40 m s^−1^ operating as a LED under different forward voltage bias. d) *EQE*_EL_ of devices prepared in ambient air by different gas blowing velocities. e) XRD patterns of perovskite thin‐films fabricated in ambient conditions by air‐knife‐assisted blading with different blowing rates, as labeled. Diffraction peaks associated with FTO are marked by an asterisk.

PL studies were conducted to evaluate the optical quality of the ambient‐processed perovskite absorbing layer for fabrication of high‐performance PV devices. **Figure** **6**a,b shows the steady‐state and TRPL spectra, respectively, for the perovskite thin‐films prepared at different drying gas flow rates. There is a systematic trend in the steady‐state PL peak intensity, in which the sample prepared by the highest drying‐gas flow rate, 40 m s^−1^, exhibits the highest PL peak intensity. This is attributed to the formation of less non‐radiative recombination centers in the bulk material and is in good agreement with the SEM images in Figure 3f, which shows an extraordinarily compact and pinhole‐free morphology. Furthermore, no PL signal could be detected from the sample dried naturally in ambient conditions at high humidity, which is in contrast with the results of our previous study on films dried naturally in a glovebox.^[^ ^12^ ^]^ A relatively strong PL signal was detected from the sample dried naturally in a glovebox, as shown in Figure S6, Supporting Information. Compared to the PL intensity of the film dried at 40 m s^−1^ in the glovebox, which was considered as 1.0 for normalization, the PL intensity of the film dried naturally in the glovebox is ≈67%, while no PL occurs for film dried naturally in high‐humidity ambient air. This indicates that long exposure in humid environments is detrimental to perovskite film quality. When the air‐knife speed increases, both the glovebox‐ and ambient‐fabricated perovskite films show an increase in PL intensity, indicating a higher quality of perovskite film for solar cells. In particular, the PL intensity of ambient‐processed perovskite films increases significantly as a function of air‐knife gas‐blowing speed than that of glovebox‐processed perovskite films. The PL intensities of the ambient processed samples were 0, 12, 63, and 100% at 0 (naturally dried case), 14, 28, and 40 m s^−1^, respectively. Thus, air‐knife‐assisted drying not only provides a highly crystalline perovskite film but also effectively suppresses moisture attack in ambient conditions with a high relative humidity of 55%.

TRPL was also conducted to examine the carrier lifetimes of the perovskite thin‐films prepared under ambient conditions at different drying gas velocities, as shown in Figure 6b. Superposition of fast and slow dynamics was observed in the TRPL data. Both the fast and slow transient components were investigated using a biexponential fitting model. The slow (and fast) carrier lifetimes for the samples under 14, 28, and 40 m s^−1^ gas‐blowing flowrates are 7.6 (and 407.9), 7.7 (and 559.6), and 7.2 (and 620.9) ns, respectively. These two significantly different time‐scaled transients are assigned as the surface (fast) and bulk (slow) components.^[^ ^86^ ^]^ The relative contributions of the fast and slow components are summarized in Table S3, Supporting Information. It was found that the slow transient component dominates, constituting over 93% of the entire carrier transient process. Thus, both the steady‐state PL and TRPL results reveal that air‐knife‐assisted drying at high blowing velocity contributes to the suppression of non‐radiative recombination centers and bulk traps.

It is known that the non‐radiative recombination process has an impact on the *V*_OC_ of a real device. The *V*_OC_ loss is a key factor influencing the maximum achievable *PCE* of the solar cell, which is the difference between *E*_g_/*q* of the photo‐absorbing layer and the *V*_OC_ of the actual device. The losses can be divided into three components. According to the detailed balance theory, the Shockley–Queisser (SQ) limit $V_{\mathrm{oc}}^{\mathrm{SQ}}$ is 1.33 eV for PSCs with an energy bandgap of 1.61 eV.^[^ ^87^, ^88^ ^]^ This voltage loss, $V_{\mathrm{oc}}^{\mathrm{SQ}},$ is unavoidable radiative recombination. The second loss, $V_{\mathrm{oc}}^{R},$ is the difference between $V_{\mathrm{oc}}^{\mathrm{SQ}}$ and *V*_OC_ when only radiative recombination occurs, which is due to the fact that the band edge is not perfectly abrupt but is defined as a step function in SQ theory.^[^ ^87^ ^]^ The performance of mixed‐halide PSCs is mainly affected by trap‐assisted non‐radiative recombination.^[^ ^89^ ^]^ Thus, to achieve high *V*_OC_ in a solar cell, the non‐radiative loss should be minimized and has been widely studied by many groups who rely on the results of the electroluminescence quantum efficiency.^[^ ^89^, ^90^, ^91^ ^]^ The non‐radiative recombination can induce *V*_OC_ losses in PSCs, and the voltage loss due to non‐radiative recombination, $\Delta V_{\mathrm{oc}}^{\mathrm{NR}}$, can be quantitatively evaluated by the relation:^[^ ^88^, ^89^, ^90^, ^92^, ^93^ ^]^

(4) $$\Delta \;V_{\mathrm{oc}}^{\mathrm{NR}}=-\frac{k_{B}T}{q}\ln\left(EQE_{\mathrm{EL}}\right)$$

where *EQE*_EL_ is the electroluminescence quantum efficiency when the injection current density in the dark is equal to the *J*_SC_ of the device under 1 sun illumination. We tested our solar cells as light‐emitting diodes (LEDs) in the dark under different voltage biases from 0 to 2.5 V. It is worth noting that the EL of the device is clearly visible in ambient lighting and is as bright as the voltage readout LED indicator, as shown in the inset of Figure 6c. Figure 6d shows the measured *EQE*_EL_ as a function of the forward injection current density for devices prepared at different gas‐blowing speeds in an ambient air environment. The ambient‐air‐processed devices exhibit a maximum *EQE*_EL_ (or *EQE*_EL_ at *J*_SC_) of 4.95% (0.794%) and 0.30% (0.022%) at a gas‐blowing rate of 40 and 28 m s^−1^, respectively. For the device prepared at the lowest gas‐blowing speed of 14 m·s^−1^, there was no detectable EL signal. Based on Equation (4), the calculated non‐radiative voltage losses at *J*_SC_ for the 40 and 28 m s^−1^ blow‐dried devices are 0.125 V and 0.217 V, respectively. It is also important to note that comparable or slightly better *EQE*_EL_ is obtained for the device prepared in ambient air than the device prepared in a glovebox, as shown in Figure S7, Supporting Information. The detailed results are summarized in Table S4, Supporting Information. Thus, our results show that the non‐radiative recombination defects can be significantly suppressed when the room‐temperature blade‐coated perovskite wet film is dried at a gas velocity of 40 m s^−1^, which is attributed to the formation of a more compact and pinhole‐free layer, as shown in Figure 3d. This also supports the effectiveness of the technique in eliminating detrimental humidity attacks on perovskite film formation. The crystallinity of the perovskite thin‐films fabricated under ambient conditions with different air‐knife blowing rates was investigated by X‐ray diffraction, as shown in Figure 6e. The diffractogram shows that the perovskite thin‐film without air‐knife blowing is comprised of different phases than the one with air‐knife blowing. Typical diffraction peaks at 14.10°, 20.02°, 23.47°, 28.42°, and 30.89° correspond to the Miller planes (110), (112), (211), (220), and (213) of the tetragonal perovskite phase^[^ ^94^ ^]^ and are present in the air‐knife‐blown samples. The higher blowing speed results in increased intensity of the typical diffraction peaks from the tetragonal perovskite phase, indicating a better crystallinity and quality of the perovskite film. Thus, these results are in good agreement with the PV performance discussed above.

**Figure 7:**
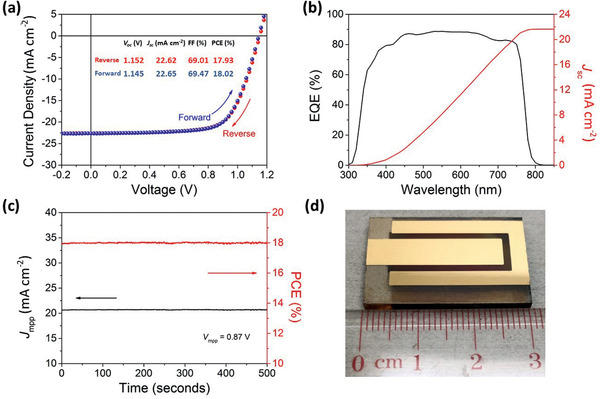
Performance of device with an area of 1 cm^2^: a) *J–V* characteristics, b) *EQE*, and c) stability test at maximum power point of d) the champion 1 cm^2^ device. The integrated current density derived from the *EQE* spectrum is 21.65 mA cm^−2^.

Furthermore, we demonstrated a large‐area PSC device with an active area size of 1 cm^2^ using this facile room‐temperature ambient‐air fabrication method. **Figure** **7**a shows the *J–V* characteristics of the champion large‐area device prepared under the same conditions as the champion cell shown in Figure 2d. The *PCE* of the 1 cm^2^ device was 18.0% with negligible hysteresis, associated with a *V*_OC_ of 1.15 V, *J*_SC_ of 22.65 mA cm^−2^, and an *FF* of 0.69. The *EQE* of the large‐area cell and the corresponding integrated *J*_SC_ are shown in Figure 7b. The calculated *J*_SC_ (21.65 mA cm^−2^) matches well with that obtained from the *J–V* curve within 5% deviation. Furthermore, a stabilized *PCE* of 18.0% under the maximum power point is achieved at a bias voltage of 0.87 V, as shown in Figure 7c. The reduction of *PCE* in large‐area devices arises mainly from the *FF* drop from 77.7% to 69%, which indicates that the drop is mainly due to the device geometry and sheet resistance of the large FTO substrate^[^ ^58^, ^95^ ^]^ rather than the perovskite film quality during scale‐up. A blade‐coated perovskite thin film on glass substrate of size up to 10 cm × 10 cm was demonstrated using laminar gas‐assisted drying, as shown in Figure S8, Supporting Information. A highly uniform film was visualized, and UV–vis absorption spectra were measured from nine different regions to characterize the perovskite film quality. The Tauc plot method was used to evaluate the bandgap energy of the perovskite film, which was found to be 1.602 ± 0.001 eV, further proving the scalability of this approach. In manufacturing, the humidity variation due to weather changes is another factor to consider. Our results show that a comparable high‐device performance is achieved for the perovskite layer prepared in ambient conditions with relative humidity up to 65%, indicating a high tolerance to humidity variation. It is worth noting that there is a significant drop in *PCE* for the devices prepared in a very humid environment (*RH* = 75%), as shown in Figure S9, Supporting Information, as expected. Many previous reports, which are summarized in Figure 1b, show a more severe efficiency drop starting at a much lower humidity level. Therefore, our work provides a much‐improved tolerance to humidity changes. These promising results demonstrate the realization of a high‐quality, ambient‐air‐processed perovskite layer by scalable meniscus‐blade coating and air‐knife‐assisted drying, paving the way for technology transfer from a sophisticated glovebox technique to low‐cost, ambient‐air scalable manufacturing at a relative humidity of as high as 55%.

## 3 Conclusion

In summary, a facile, scalable perovskite fabrication method at room temperature under high‐humidity ambient conditions was demonstrated for creating efficient PSCs based on the highly developed, champion perovskite solution system DMF–DMSO. The synergetic effect of the air knife, which provides protection against oxygen and water molecules, combined with the accelerated evaporation rate of solvents in the precursor solution, enables efficient PSC fabrication in air. This laminar air‐knife‐assisted perovskite film formation process under ambient conditions was quantitatively investigated by utilizing a unique in situ TR UV–vis spectrometry technique. The gas‐blowing speed and consequent solvent removal rate play an important role in determining the degree of super‐saturation, which affects the perovskite crystal nucleation and crystal growth process, thus affecting the morphology and PV performance of the perovskite film. This ambient fabrication approach results in PSCs with *PCE*s up to 21.1%, a stabilized *PCE* of 20.6% for small active area (0.06 cm^2^), and a *PCE* of 18.0% for large‐area devices (1 cm^2^) with negligible hysteresis. Furthermore, a maximum shelf‐life stability (*RH* = 30% at room temperature) of 95% of the initial *PCE* over 80 days was obtained for the PSCs fabricated in ambient conditions and their glovebox‐fabricated counterparts, indicating the high potential of this technology.

## 4 Experimental Section

##### Materials Preparation

Formamidine iodide (FAI) and methylammonium bromide (MABr) were purchased from Dyesol. Lead(II) bromide (PbBr_2_), methylammonium chloride (MACl), cesium iodide (CsI), rubidium iodide (RbI), potassium iodide (KI), bis(trifluoromethane) sulfonimide lithium salt (Li‐TFSI), tBP, acetonitrile (ACN), chlorobenzene (CB), DMF, and DMSO were purchased from Sigma Aldrich. Tin(IV) oxide (15% in H_2_O colloidal dispersion), tin(II) chloride dihydrate, and 2‐(2‐aminoethyl)isothiourea dihydrobromide were purchased from Alfa Aesar. Lead(II) iodide (PbI_2_) was purchased from TCI, and spiro‐OMeTAD was purchased from Lumtec. All chemicals were used as received, without further purification. The SnO_2_ quantum dot (QD) solution was synthesized by dissolving SnCl_2_·2H_2_O and 2‐(2‐aminoethyl)isothiourea dihydrobromide in a molar ratio of 1:6.5 in 30 mL deionized water under vigorous stirring with continuous oxygen flowing at room temperature. The SnO_2_ nanoparticle (NP) solution was prepared by mixing tin(IV) oxide (15% in H_2_O colloidal dispersion) and de‐ionized water with a volume ratio of 1:5. A perovskite precursor solution (46 wt% solute:solution) was prepared by dissolving FAI:MABr:MACl:PbBr_2_:PbI_2_ in a molar ratio of 1.1:0.2:0.5:0.2:1.2 into a mixed solvent system of DMF and DMSO (4:1, v/v) with 1.5 m CsI (2.8%, by volume) and 1.5 m RbI (2.8%, by volume) and 1.5 m KI (1.8%, by volume) in a nitrogen‐filled glovebox. The hole‐transport‐layer solution was prepared by dissolving spiro‐OMeTAD (80 mg) in 1 mL CB with 29 µL of tBP and 17.5 µL of Li‐TFSI (520 mg·mL^−1^ in ACN).

##### Device Fabrication

PSCs based on ambient‐air room‐temperature meniscal‐coated perovskite layers were fabricated with a device structure of FTO/SnO_2_ QDs/SnO_2_ NPs/perovskite/spiro‐OMeTAD/Au electrode, as shown in Figure 2a. Patterned glass/FTO substrates (OPVTECH, China) were ultrasonically cleaned using detergent, followed by sequential ultrasonic bath with deionized water, acetone, and isopropanol for 20 min and then blown dry with filtered N_2_. The substrates were further treated with UV ozone for 30 min to remove organic residues before device fabrication. The spin coating and meniscus blading of the SnO_2_ ETL and perovskite precursor solution, respectively, as well as post‐annealing were performed under ambient air conditions with a relative humidity of 55 ± 5% at room temperature (25 °C). Electron transport bilayers were first prepared by spin coating a SnO_2_ QD solution at a spin speed of 4000 rpm for 30 s, followed by annealing at 150 °C for 30 min. Then, the SnO_2_ NP solution was spin coated at the same spin speed as that of the SnO_2_ QDs solution, followed by heat treatment at 200 °C for 60 min. Ambient‐air‐processed meniscus‐blade coatings of perovskite photoactive layers were then coated on SnO_2_ ETLs. The gap between the coating blade and substrate was set at 90 µm, and the coating speed was maintained at ≈600 mm·min^−1^. The as‐bladed wet films were then subjected to air‐knife‐assisted drying at different blowing velocities. Filtered N_2_ gas was used at velocities of 0, 14, 28, and 40 m s^−1^ to accelerate solvent evaporation and crystallization. The gas flow rate was measured using a Testo 416 flowmeter. Next, the blow‐dried perovskite thin‐films were annealed in air at 120 °C for 60 min. The samples were then transferred to a N_2_‐filled glovebox to perform the spiro‐OMeTAD spin coating at 4000 rpm for 60 s. Finally, the top metal electrodes were formed by thermal evaporation of an 80 nm thick Au layer using shadow masks. The active area of the device was 0.06 cm^2^. For the large‐area device, the active area was 1 cm^2^, which was defined by the shadow mask of size 2 × 0.5 cm^2^.

##### Characterization

The in situ UV–vis spectra were measured using an F20 spectrometer (Filmetrics, Inc.). The light source was a halogen lamp with a spot size of 1.5 mm. A blank FTO‐coated glass substrate was used as the reference so that the measured signal revealed the absorption or transmission of the perovskite thin film. The in situ transmission signal was recorded to monitor the uniformity and thickness of the as‐bladed wet film, as discussed in Figure 3a. The absorbance of the thin film was evaluated by *A* = 2 − log_10_ *(T*), where *T* is the transmission of the thin film, which provides a unique in situ characterization of the drying kinetics and crystallization rate of the perovskite thin‐film under ambient air conditions. The crystal structure was characterized using a 9 kW Rigaku SmartLab X‐ray diffractometer with Cu K*α* radiation utilizing *θ*–2*θ* scan mode from 10° to 70°. The thin‐film morphology was characterized by field‐emission SEM using a JEOL JSM‐6335F. TRPL studies of the perovskite thin‐films on quartz substrates were measured using an Edinburgh FLSP920 spectrophotometer equipped with an excitation source of 485 nm picosecond pulsed diode laser with an average power of 0.15 mW. Data acquisition and data fitting were performed using the FLSP920 system software. The *I–V* characteristics of the devices were measured using a Keysight B2901A source meter under a calibrated solar simulator with an AM 1.5 filter at 100 mW cm^−2^ (Enli Technology Co. Ltd.). The *I–V* curves were measured by scanning from 1.2 V to −0.2 V with a delay time of 50 ms. The corresponding *EQE* was measured by a QE‐R3011 system from Enli Technology Co., Ltd. An Si reference detector with NIST‐traceable certification was used for power calibration before measurement. The *EQE* measurements were performed using the AC mode under monochromatic light split from a 75 W Xe lamp from 300 to 900 nm. The electroluminescence *EQE* was measured using a commercial quantum yield measurement system (LQ‐100X) equipped with an integrating sphere from Enli Technology Co., Ltd.

## Conflict of Interest

The authors declare no conflict of interest.

## Supporting information

Supporting Information
