# Supplementary material for: Printing High‐Efficiency Perovskite Solar Cells in High‐Humidity Ambient Environment—An In Situ Guided Investigation
Source: Adv Sci (Weinh). 2021 Jan 25;8(6):2003359. doi: 10.1002/advs.202003359 (PMC7967091; doi:10.1002/advs.202003359)
Supplement: Supplementary file 1 — Supporting Information [file ADVS-8-2003359-s001.pdf]

WILEY-VCH

© 2020 Wiley-VCH GmbH

ADVANCED SCIENCE
Open Access

Supporting Information

for *Adv. Sci.,* DOI: 10.1002/advs.202003359

Printing High-Efficiency Perovskite Solar Cells in High-Humidity Ambient Environment—An In-situ Guided Investigation

*Patrick Wai-Keung Fong, Hanlin Hu, Zhiwei Ren, Kuan Liu, Li Cui, Tao Bi, Q. Liang, Zehan Wu, Jianhua Hao, and Gang Li**

Supporting Information

Printing High-Efficiency Perovskite Solar Cells in High-Humidity Ambient Environment—An In-situ Guided Investigation

*Patrick Wai-Keung Fong, Hanlin Hu, Zhiwei Ren, Kuan Liu, Li Cui, Tao Bi, Q. Liang, Zehan Wu, Jianhua Hao, and Gang Li**

Blowing gas velocity [$m \cdot s^{-1}$]	V_{OC} [V]	J_{SC} [mA cm^{-2}]	FF [%]	PCE [%]
0 (ambient air)	N/A	N/A	N/A	N/A
14 (ambient air)	0.71±0.22	12.53±2.81	38.14±8.96	3.77±2.13
28 (ambient air)	1.03±0.05	19.59±0.96	57.71±8.07	11.66±2.01
40 (ambient air)	1.16±0.01	22.01±0.82	74.83±2.23	19.16±0.90
40 (control, glovebox)	1.17±0.01	21.84±0.89	73.82±2.56	18.85±0.93

Table S1. The photovoltaic performance of ambient-air-processed PSCs with air-knife-assisted drying at different blowing velocities. The control is fabricated with air-knife-assisted drying within the glovebox.

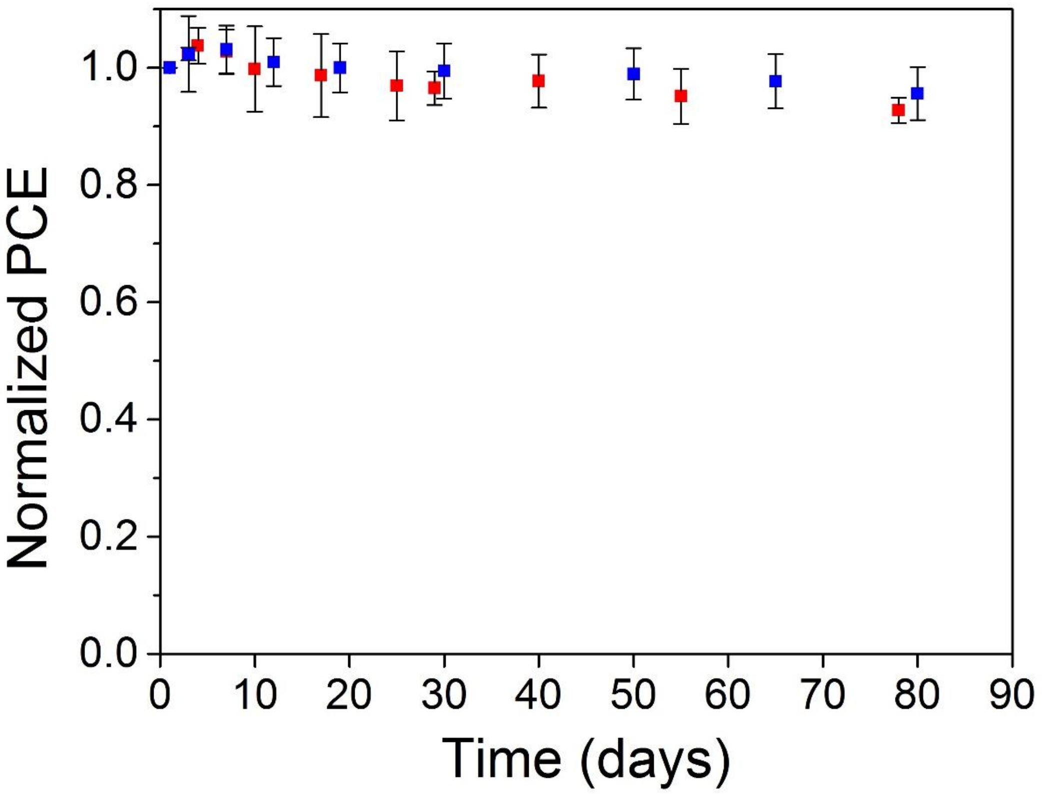

Figure S1. Shelf-life stability test of four ambient-air-processed devices (0.06 cm^2) prepared by air-knife-assisted drying at a blowing velocity of 40 $m \cdot s^{-1}$ in air (red) and a glovebox (blue). The devices are unencapsulated and stored in a dry box of RH = 30% at room temperature.

Supporting Note 1:

The air stabilities of four unsealed devices prepared by air-knife-assisted drying at a blowing velocity of 40 $m \cdot s^{-1}$ under ambient air conditions (red) and in an inert-gas-filled glovebox (blue) were measured, as shown in Figure S1. The devices were stored in an ambient environment at 25 °C with a relative humidity of 30%. Excellent shelf-life stability, with an average PCE remained at 95% for up to 80 days, was obtained for both the ambient-air-processed and glovebox-processed perovskite photo-absorbing layer, indicating that the quality of the ambient-air-processed-devices is comparable to that of those processed in a glovebox.

Supporting Note 2:

We demonstrated the process control of thickness and uniformity of the blade-coated wet film by in-situ UV-vis spectrometry. A sinusoidal interference pattern superimposed on the transmission spectrum was observed when the thickness of the wet film was uniform. In case of non-uniform thickness or in the presence ripples, the interferences pattern disappeared. Interference fringes with thickness (d_f) and refractive index (n_f) of the wet film are formed when:

$$2n_f d_f = m\lambda, \qquad \text{(S1)}$$

where m is an integer for maxima order and λ is the wavelength. Figure 3a shows the transmission spectra, exhibiting interference oscillations, of the as-bladed wet film prepared in an ambient air environment for two different samples. There are two independent variables in **Equation S1**, and d_f cannot be evaluated if n_f is unknown. Although n_f and d_f are both unknown, the thickness of the wet film can be compared from the slope of a plot of $1/\lambda$ versus the maxima order, as shown in Figure 3b. Nearly parallel slopes were observed for the two samples, demonstrating that the two wet films are of equal thickness. The two independent variables (n_f and d_f) can be evaluated using the model of envelope confining the interference fringes, where T_M is the upper bound and T_m is the lower bound. From the interference fringes, the upper bound can be expressed as

$$T_M = \frac{2n_s}{{n_s}^2+1}, \qquad \text{(S2)}$$

where n_s is the refractive index of the substrate. The lower bound is given by

$$T_m = \frac{4{n_f}^2 n_s}{{n_s}^2+{n_f}^2({n_s}^2+1)+{n_s}^2}\,. \qquad \text{(S3)}$$

Rearranging **Equation S2** and **S3**, the refractive index of the wet film is

$$n_f = \sqrt{2n_s \frac{T_M - T_m}{T_M T_m} + \frac{n_s^2+1}{2} + \sqrt{\left(2n_s \frac{T_M - T_m}{T_M T_m} + \frac{n_s^2+1}{2}\right)^2 - n_s^2}} \,. \qquad \text{(S4)}$$

If n_{f1} and n_{f2} are the refractive indices at two adjacent maxima or minima of the interference fringes at λ_1 and λ_2, respectively, then by solving Equations S1 and S4, the thickness of the wet film can be evaluated as

$$d_f = \frac{\lambda_1 \lambda_2}{2(\lambda_1 n_{f_1} - \lambda_2 n_{f_2})} \,. \qquad \text{(S5)}$$

Hence, from the upper and lower bounds of the interference fringes, the thickness of the as-bladed wet films was estimated as 2.6 µm.

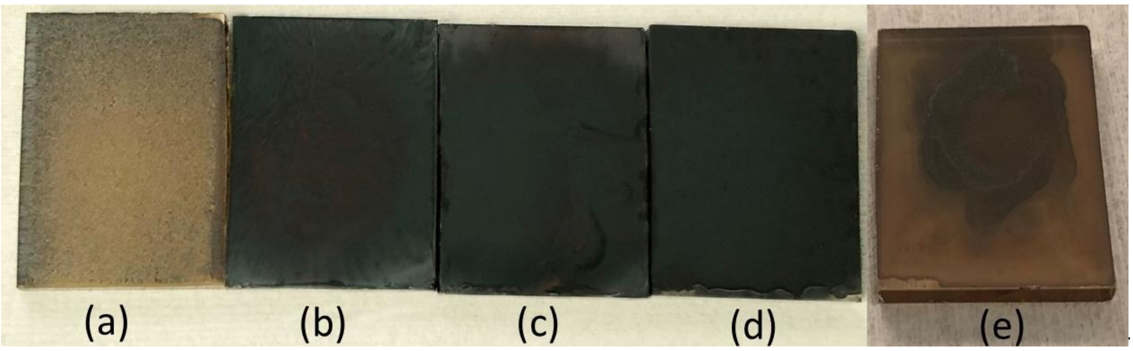

Figure S2. Photographs of the ambient-air meniscus-blade-coated perovskite thin films (2 cm by 3 cm) being dried by air knife at blowing gas velocities of 0, 14, 28, and 40 m·s^{-1}, as shown in (a), (b), (c), and (d), respectively, and e) the thermally annealed sample without air-knife-assisted drying.

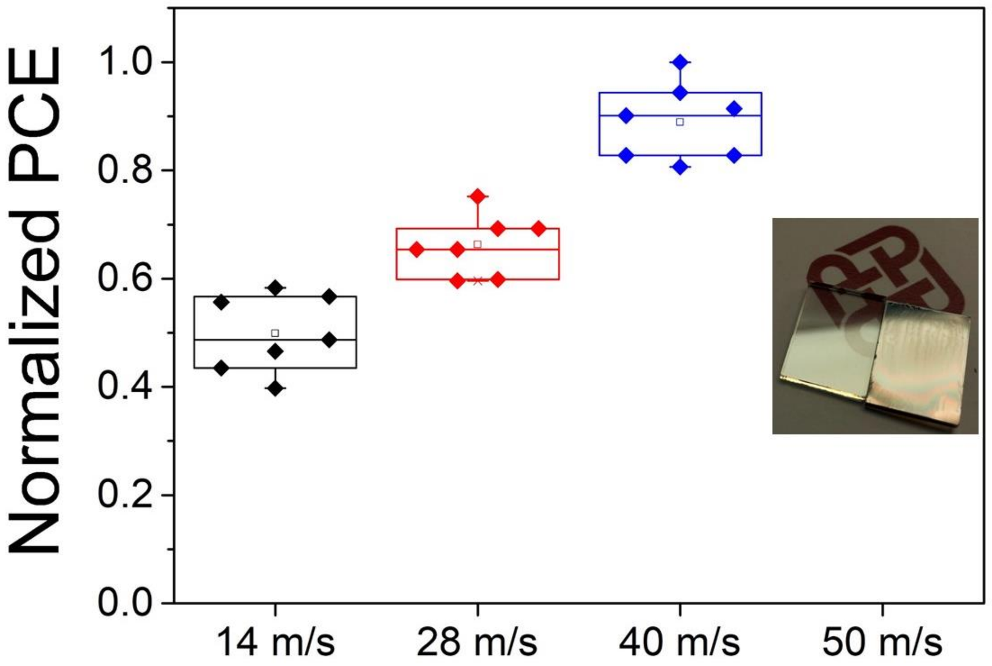

Figure S3. Normalized power conversion efficiency for the devices prepared by ambient-air meniscus-blade-coated perovskite precursor of 36 wt% concentration being dried by air knife at different blowing gas velocities. A photograph shows the spreading of wet film (on the right) at a gas velocity of 50 $m \cdot s^{-1}$.

Supporting Note 3:

Device performance (precursor concentration = 36 wt% solute:solution) increases as the blowing gas velocity increases (Figure S3), which is consistent with the devices prepared by a precursor solution concentration of 46 wt% (Figure 2b). The optimal blowing gas velocity is expected to be dependent on the concentration of the precursor solution. However, our results show that the shear stress on the wet film is the limiting factor; similar observations were reported by Hu et al.[1] Scale-like features are observed, indicative of solution flow due to the strong force by high gas velocity at 50 $m \cdot s^{-1}$, as shown in Figure S3. A similar phenomenon was reported in the air-bladed perovskite layer, in which a large gas blow force was employed to spread the perovskite precursor solution on the substrate.[2] Notably, the viscosity of 46 wt% and 36 wt% solute in the precursor solution are very close. The viscosity varied from

1.59 (at 30 wt%) to 2.00 (at 40 wt%) mPa·s for mixed-halide perovskite solution at various concentrations.[3] For reference, the viscosities of pure H_2O and pure DMSO are 0.89 and 1.99 mPa·s, respectively, at 25 °C.[4] Thus, we believe that the difference in viscosity is too small, and the optimal gas-blowing velocity at different concentrations of the precursor solution is not distinguishable within experimental error.

[1] H. Hu, Z. Ren, P. W. K. Fong, M. Qin, D. Liu, D. Lei, X. Lu, G. Li, Advanced Functional Materials **2019**, 29, 1900092.

[2] J. Ding, Q. W. Han, Q. Q. Ge, D. J. Xue, J. Y. Ma, B. Y. Zhao, Y. X. Chen, J. Liu, D. B. Mitzi, J. S. Hu, Joule **2019**, 3, 402.

[3] M. Habibi, A. Rahimzadeh, I. Bennouna, M. Eslamian, Coatings **2017**, 7, 42.

[4] R. G. LeBel, D. A. I. Goring, Journal of Chemical & Engineering Data **1962**, 7, 100.

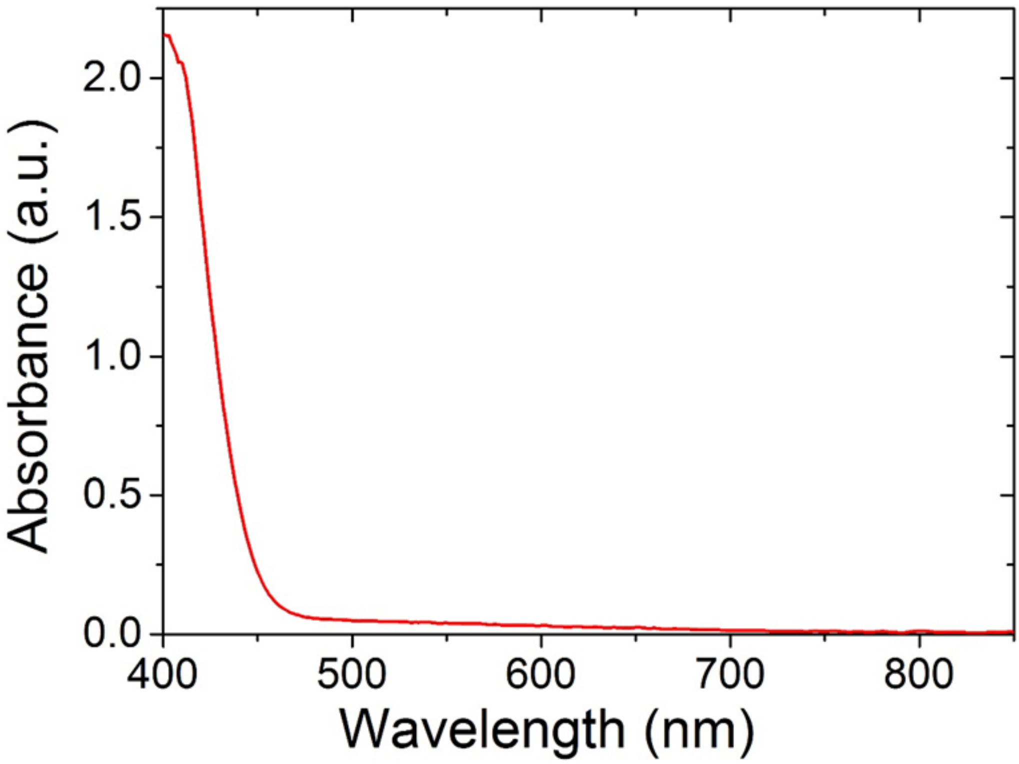

Figure S4. Absorption spectrum of the precursor solution measured by in-situ UV-vis spectrometry.

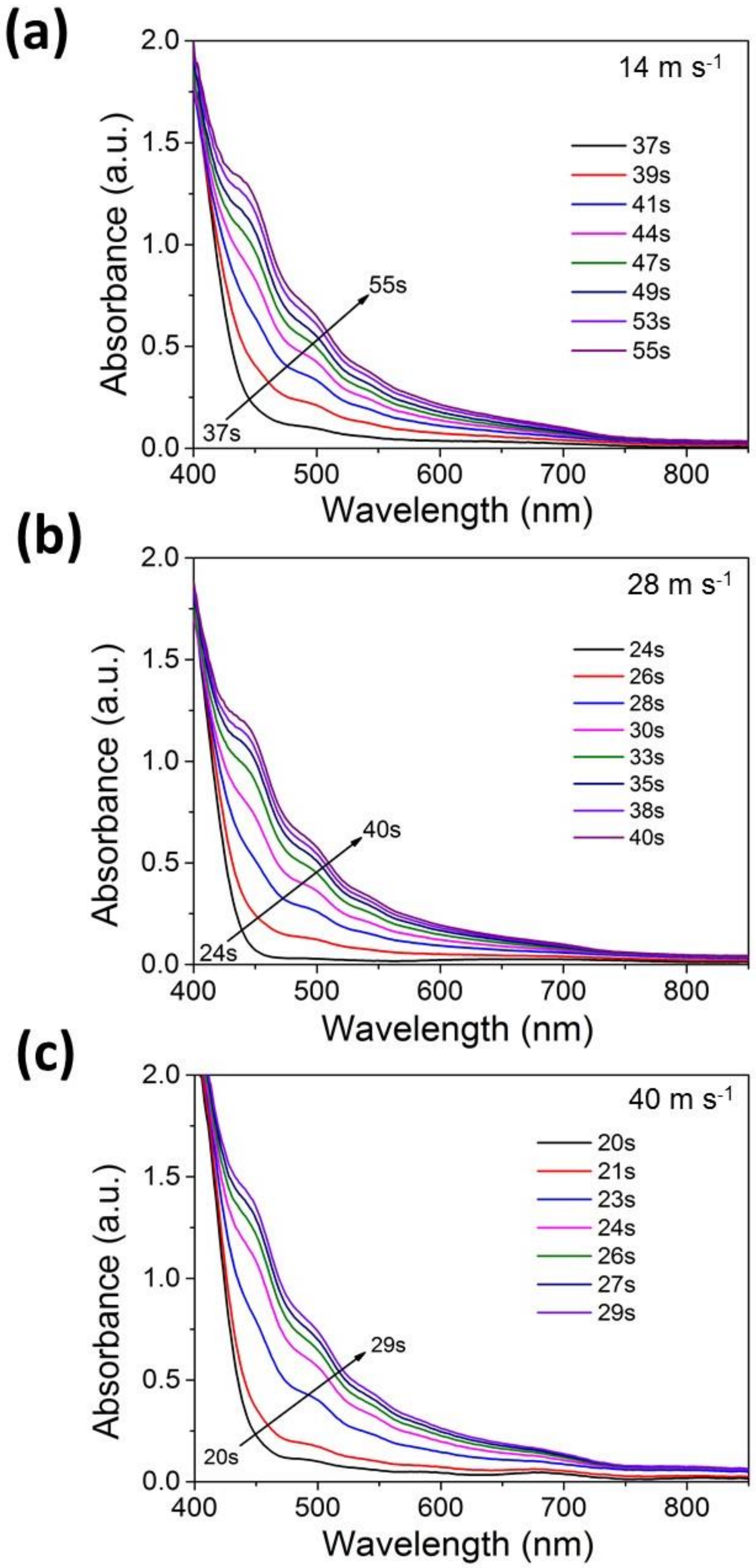

Figure S5. Evolution of intermediate-state absorbance spectra for the samples prepared by air-knife-assisted drying at different gas velocities of a) 14 $m \cdot s^{-1}$, b) 28 $m \cdot s^{-1}$, and c) 40 $m \cdot s^{-1}$, respectively.

Gas blowing velocity<br>[m·s^{-1}]	*Max. dA/dt*<br>at 450 nm [s^{-1}]<br>(norm. value)	*Max. dA/dt*<br>at 550 nm [s^{-1}]<br>(norm. value)	*Max. dA/dt*<br>at 650 nm [s^{-1}]<br>(norm. value)
14	0.097 (0.37)	0.031 (0.40)	0.012 (0.36)
28	0.127 (0.49)	0.036 (0.47)	0.014 (0.43)
40	0.259 (1)	0.076 (1)	0.033 (1)

Table S2. Calculated peak absorbance change rate, max. *dA/dt*, at λ = 450, 550, and 650 nm for the wet perovskite film prepared under different gas-blowing velocities. The normalized values are shown in parentheses for comparison.

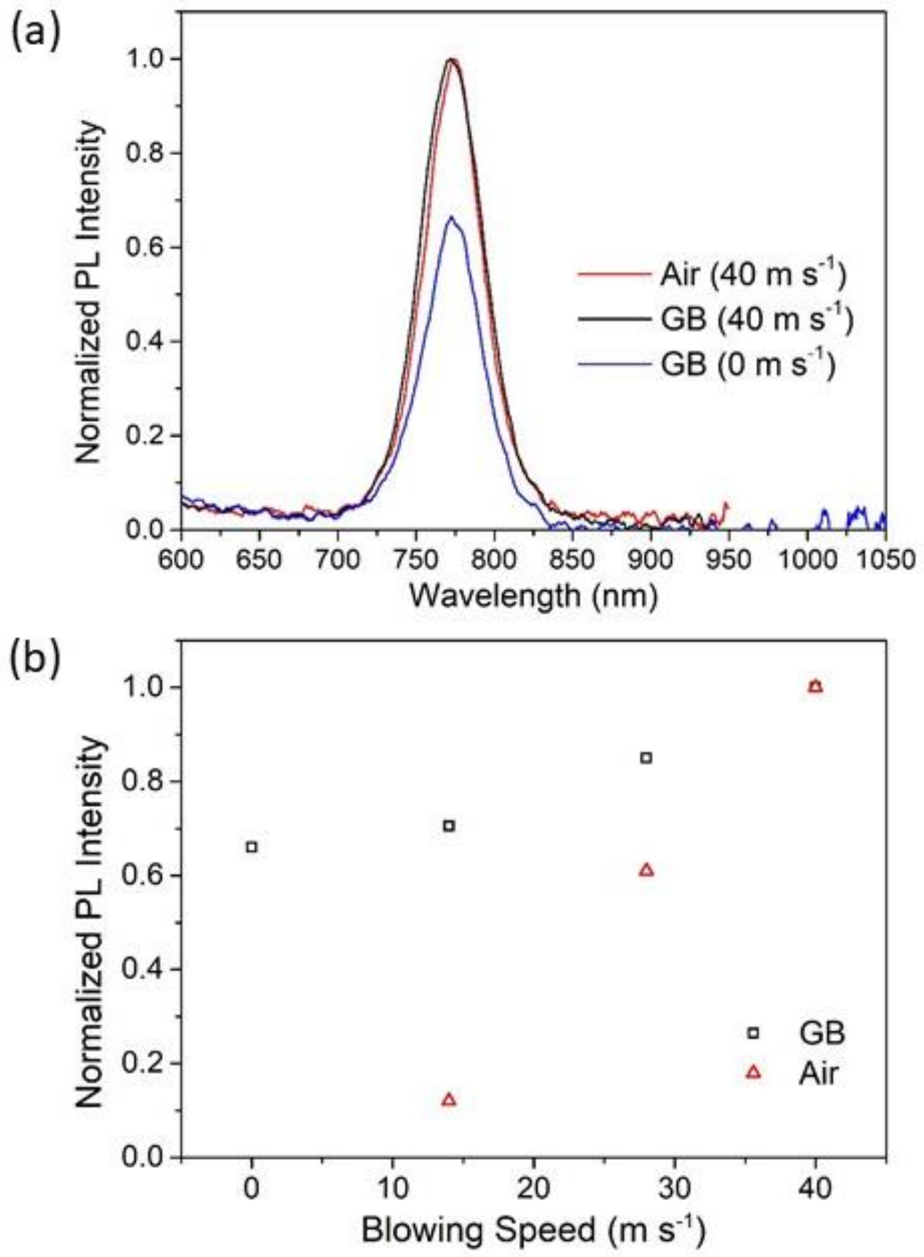

Figure S6. a) Steady-state PL spectra for the samples prepared by air-knife-assisted drying at 40 m·s^{-1} in an ambient environment and in an inert-gas-filled glovebox (GB). The naturally dried (0 m s^{-1}) sample is shown to indicate the impact of moisture attack. b) The relative PL intensity for samples dried at different gas-blowing velocities in ambient air and glovebox environment. There is no PL signal detected from the sample dried naturally in ambient air.

Gas blowing velocity [m·s^{-1}]	τ_1 [ns] (contribution)	τ_2 [ns] (contribution)
14	7.6 (6.7%)	407.9 (93.3%)
28	7.7 (4.6%)	559.6 (95.4%)
40	7.2 (6.5%)	620.9 (93.5%)

Table S3. Carrier lifetime fitted from the TRPL data using a biexponential model. The contribution percentage is shown to reveal the weighted contribution of the corresponding carrier transient component.

Supporting Note 4:

The carrier lifetime was evaluated by using the biexponential PL decay model, which can be expressed as:

$$R(t) = B_1 \exp(-t/\tau_1) + B_2 \exp(-t/\tau_2), \qquad \text{(S6)}$$

where B_i and τ_i are the amplitude and decay time constants of the i-th component, respectively.

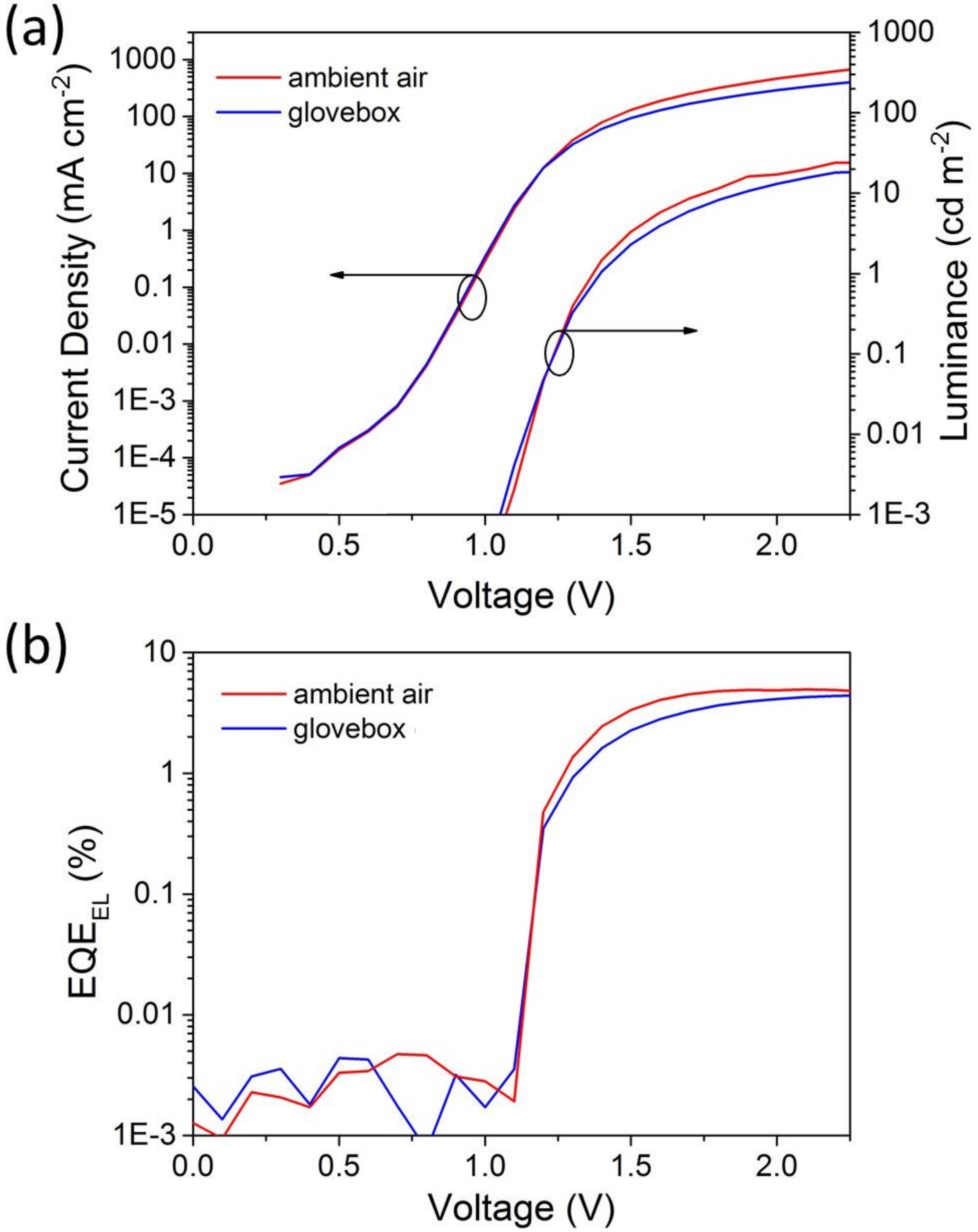

Figure S7. a) Plot of current density and luminance as a function of bias voltage for the ambient-air-processed and glovebox-processed (control) devices at a gas blowing velocity of 40 m·s^{-1} and b) the corresponding EQE_{EL}.

Gas blowing velocity [m·s^{-1}]	V_{OC} [V]	EQE_{EL} max. [%]	EQE_{EL} at J_{SC} [%]	ΔV_{oc}^{NR} [V]
14	0.682	Undetectable	Undetectable	Undetectable
28	1.073	0.30	0.022	0.217
40	1.176	4.95	0.794	0.125
40 (control)	1.169	4.50	0.627	0.131

Table S4. A summary of the EL parameters of the ambient-air-processed perovskite layers prepared by air-knife-assisted drying at different gas blowing velocities. The control was prepared at the same experimental condition, but inside the glovebox.

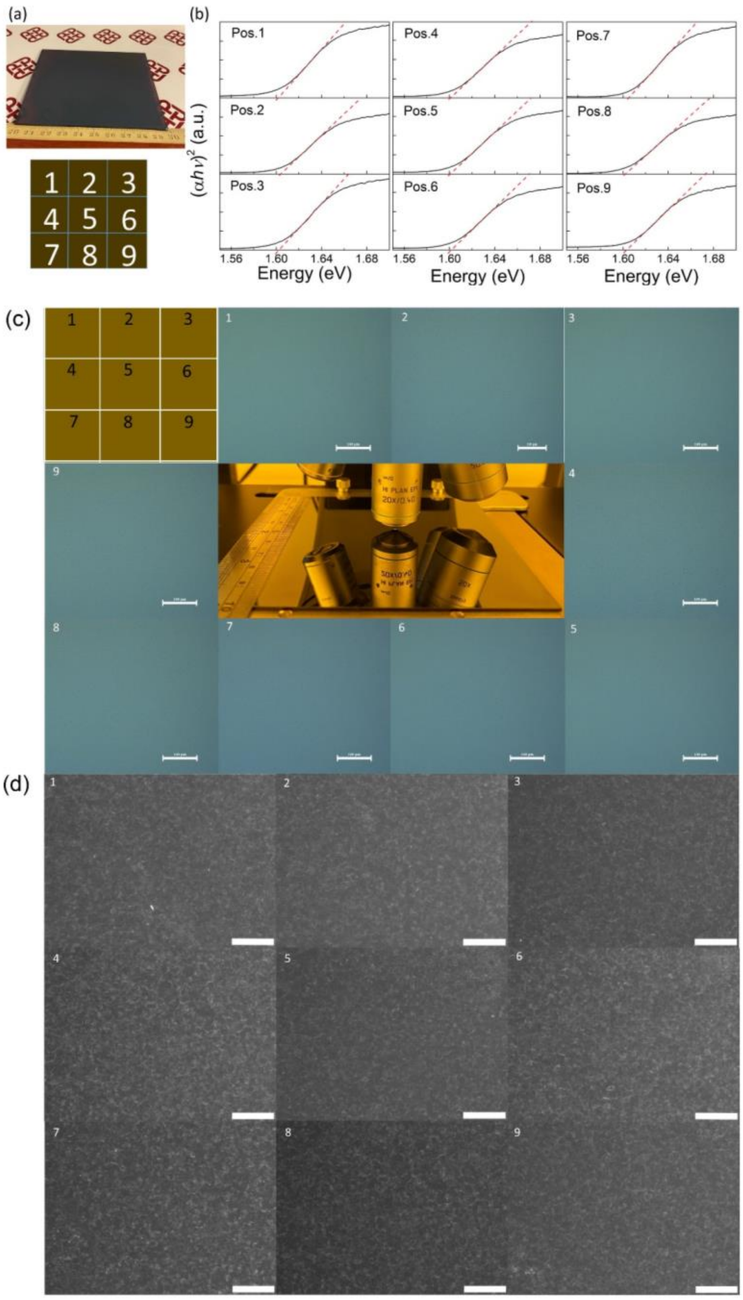

Figure S8. a) Photograph of the ambient-air meniscus-blade-coated perovskite thin-film (10 cm × 10 cm) prepared by laminar-air-knife-assisted drying and a schematic diagram showing the numbering of nine different regions characterized by UV-vis absorption spectroscopy. b) The corresponding Tauc plots measured from nine different regions to evaluate the bandgap energy, which is calculated to be 1.602±0.001 eV. c) Optical and d) SEM images taken from nine different regions (scale bars for optical and SEM images are 100 μm and 5 μm, respectively).

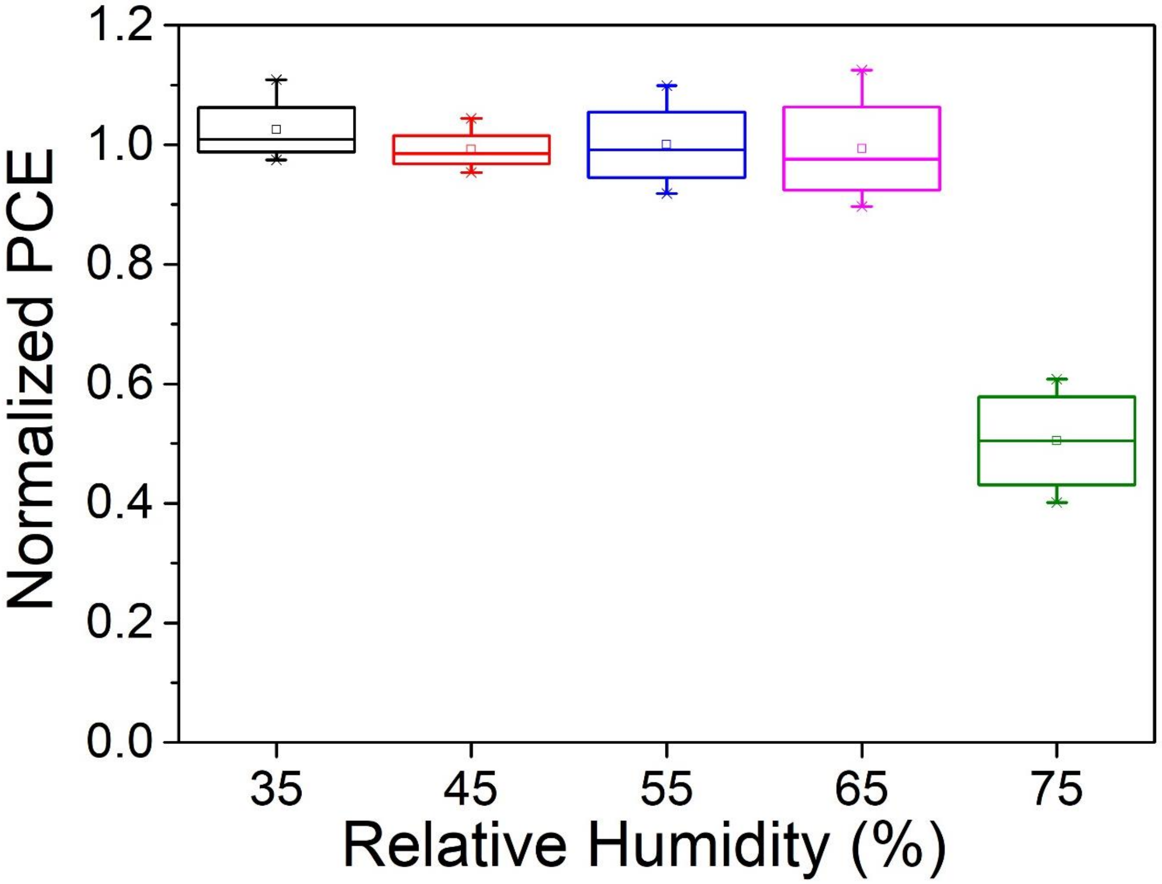

Figure S9. Normalized (at a mean PCE of 55% RH) power conversion efficiency of perovskite solar cells with the meniscus-blade-coated perovskite active layer prepared in ambient air at different relative humidities.
